# Frequency of antidepressant use and clinical characteristics of children and adolescents undergoing polysomnography: an observational study

**DOI:** 10.1186/s13034-023-00599-7

**Published:** 2023-04-29

**Authors:** Lourdes M. DelRosso, Oliviero Bruni, Maria P. Mogavero, Amy Fickensher, Carlos H. Schenck, Raffaele Ferri

**Affiliations:** 1 Pulmonary and Sleep Medicine, University of California San Francisco-Fresno, Fresno, CA USA; 2 Department of Social and Developmental Psychology, Sapienza University, Rome, Italy; 3 Vita-Salute San Raffaele University, Milan, Italy; 4 Sleep Disorders Center, Division of Neuroscience, San Raffaele Scientific Institute, Milan, Italy; 5 Seattle Children’s Hospital, Seattle, WA USA; 6 Minnesota Regional Sleep Disorders Center, Department of Psychiatry, Hennepin County Medical Center, University of Minnesota Medical School, Minneapolis, MN USA; 7 Oasi Research Institute – IRCCS, via C. Ruggero 73, Troina, 94018 Italy

**Keywords:** Children, Adolescents, Antidepressants, Sleep, Polysomnography, Periodic leg movements during sleep, Selective serotonin reuptake inhibitors, Serotonin and norepinephrine reuptake inhibitors, Tricyclic antidepressants, Atypical antidepressants

## Abstract

**Background:**

Antidepressants are increasingly used in children for various psychiatric disorders but also for sleep disorders such as insomnia; however, it is currently unknown how many children undergoing polysomnography (PSG) are taking anti-depressants. The aims were: to determine the frequency of use of antidepressants in paediatric patients referred for PSG, to identify the most common antidepressants used, to investigate the reasons for their use, and to analyse the PSG parameters found in children taking antidepressants.

**Method:**

An observational cross-sectional retrospective chart review of all children undergoing PSG at Seattle Children’s Hospital from 6/14/2020 to 12/8/2022 was carried out. Clinical features (such as diagnosis, especially psychiatric), sleep disorders (such as insomnia and restless sleep), and class of antidepressant used [selective serotonin reuptake inhibitors (SSRIs), serotonin and norepinephrine reuptake inhibitors (SNRIs), tricyclic antidepressants (TCA), or atypical antidepressants], and PSG parameters were collected for further analysis.

**Results:**

Among 3,371 patients who underwent PSG during the study, 367 children were selected who were taking one antidepressant only (154 boys and 213 girls, mean age was 13.7 ± years 3.69). A significantly decreased sleep stage N3 was found in girls, who were older than boys. Children with insomnia had longer sleep latency than children without, but more N3. There was a prolonged rapid eye movement (REM) sleep latency in children with attention-deficit/hyperactivity disorder and children with autism. REM latency was longer and REM percentage smaller in children taking SNRIs. Periodic leg movement index ≥ 5/hour was found in a higher number of children taking SSRIs or SNRIs (24.9%) than in subjects taking TCA or atypical antidepressants (13.3%) (chi-square 5.29, p = 0.013).

**Conclusions:**

Child and adolescent psychiatrists should question about the effects on sleep (both positive and negative) after initiating therapy with antidepressant medications.

## Background

Antidepressants are increasingly used in children for various psychiatric disorders but also for sleep disorders such as insomnia. Anxiety is one of the most common diagnoses for antidepressant use in children with a prevalence 7.2% [1], followed by major depressive disorder with a prevalence of 3.2% [1]. Both diagnoses are often comorbid [1]. Selective serotonin reuptake inhibitors (SSRIs) are the antidepressants most frequently used in children [2]. Previous studies have demonstrated that some antidepressants, particularly SSRIs, have an effect on leg movements during polysomnography (PSG), not only in adults but also in children [3]. This may be secondary to a serotonin-mediated decrease in monoaminergic inhibitory activity over the brainstem and spinal cord during sleep, especially REM sleep [4]. Similarly, chin muscle tone during rapid eye movement (REM) sleep can be pharmacologically increased, possibly interfering with the scoring of this sleep stage. Due to these effects on movements and muscle tone, the American Academy of Sleep Medicine (AASM) recommends withdrawing antidepressants several weeks prior to performing a PSG study for the diagnosis of sleep disorders that depend on the identification of REM sleep for diagnosis (e.g., narcolepsy) [5]. Furthermore, studies in adults have demonstrated that antidepressants diminish REM sleep and prolong REM latency [6]. Due to the bidirectional relationship between sleep disorders and depression/anxiety, the prevalence of depression and anxiety is higher in children with sleep disorders: 35% anxiety and 32% depression in children with obstructive sleep apnoea [7], 29.1% mood disturbances and 11.5% anxiety in children with RLS [8]. Although the risk of depression/anxiety is higher in children with sleep disorders [9], the risk of developing sleep disturbance is also higher in children with depression and anxiety [10]. It is therefore of utmost importance to understand the PSG changes in children with psychiatric disorders who are taking antidepressant medicine, as it is currently unknown how many children undergoing PSG are taking anti-depressants. This knowledge can help us understand PSG changes and establish clinical protocols in these patients.

In this study our aims were to determine the frequency of use of antidepressants in paediatric patients referred for PSG, to identify the most common antidepressants used in children referred for PSG, to investigate the reasons for the use of antidepressants, and to analyse the PSG parameters found in children taking antidepressants taking into consideration other factors or covariables that can produce these effects on the PSG.

## Methods

This was an observational cross-sectional retrospective study, which involved a chart review of all children undergoing PSG at Seattle Children’s Hospital from 6/14/2020 to 12/8/2022. Inclusion criteria included any child currently taking antidepressants at the time of the study. Data collected included: age, sex, antidepressant name, dose, psychiatric diagnosis, and PSG data (sleep latency, sleep efficiency, total sleep time, percent of time spent in non-REM sleep stages N1, N2, N3 and REM sleep, REM latency, obstructive apnoea/hypopnea index, central apnoea index, periodic limb movement during sleep [PLMS] index). Exclusion criteria included studies with less than six hours of recording, studies with artefacts that did not allow identification of sleep stages.

### Polysomnography

PSG was performed according to AASM criteria [11] overnight, in an outpatient laboratory. Patients were allowed to sleep “*ad libitum*”. Data were recorded using the Sandman Elite Natus system. Parameters recorded included electroencephalogram (two frontal, two central, and two occipital channels, referred to the contralateral mastoid); electro-oculogram, electromyogram of the submentalis muscle, electromyogram of the right and left tibialis anterior muscles, respiratory signals, effort signals for thorax and abdomen, oximetry, capnography, a single-lead electrocardiogram, video and audio recording. Calibrations were performed per routine standard by technicians. Sleep stages and leg movements were scored by a certified sleep technologist and board-certified sleep physician according to the AASM criteria [11].

### Statistical analysis

For the statistical analysis of data, beside the descriptive approach, we checked for the simultaneous effect of a series of independent factors/predictors (age, sex, presence of insomnia, diagnosis of attention-deficit/hyperactivity disorder or autism, psychiatric diagnosis and antidepressant treatment class) all known to influence sleep PSG parameters (dependent variables), by means of the General Linear Model module of the STATISTICA version 6 StatSoft Inc., Tulsa, OK. This module allows to build models for design with both categorical and continuous predictor variables. For each dependent variable, the statistical significance of the effect of the independent factors/predictors was obtained by taking into consideration the effect of the other independent factors and the univariate statistical analysis results were taken into consideration. For the analysis of frequencies, the chi-square test was used, as none of the expected frequencies in the frequency tables was below 5. The significance level was set at p < 0.05.

## Results

The total number of patients who underwent PSG during the study was 3,371 among whom 400 were taking at least one antidepressant medication; this represents 11.9% of children undergoing a PSG study. The most common reasons for referral to PSG included snoring, frequent awakenings and restless sleep. Thirty-three children were taking more than one antidepressant and were excluded from the subsequent analysis (in order to avoid uncertainty on the assignment of such subjects to an antidepressant category). Thus, the remaining 367 children were taking one antidepressant only. Their mean age was 13.7 years (standard deviation 3.69). One-hundred-and-fifty-four where male (42%) and 213 (58%) were female. Table 1 summarizes antidepressant use; the most frequently used antidepressant was sertraline, followed by fluoxetine and trazodone. Table 2 summarizes the most common diagnosis seen in this group of children: slightly more than half of them had anxiety, one third depression, and a quarter had both depression and anxiety. Attention-deficit/hyperactivity disorder (ADHD) was reported in 26.7% and 11.4% had a diagnosis of autism spectrum disorder (ASD); 3.5% reported a suicide attempt in the past. Diagnosis was per patient clinical chart as documented by the psychiatrist/primary care physician who prescribed the antidepressant.

**Table 1:** Frequency of antidepressant use

Antidepressant	N	Percentage
Sertraline	112	30.5
Fluoxetine	103	28.1
Trazodone	50	13.6
Escitalopram	22	6.0
Mirtazapine	17	4.6
Citalopram	13	3.5
Duloxetine	12	3.3
Bupropion	11	3.0
Venlafaxine	9	2.5
Amitriptyline	8	2.2
Fluvoxamine	3	0.8
Desvenlafaxine	2	0.5
Lithium	2	0.5
Nortriptyline	2	0.5
Paroxetine	1	0.3
*total*	*367*	*100*

**Table 2:** Frequency of diagnosis

Diagnosis	N	Percentage
Anxiety	186	50.7
Depression	121	33.0
Attention-deficit/hyperactivity disorder	98	26.7
Depression & anxiety	87	23.7
Autism spectrum disorder	42	11.4
Migraine/headache	33	9.0
Epilepsy	22	6.0
Suicide attempt	13	3.5
Post-traumatic stress disorder	13	3.5
Obesity	13	3.5
Tourette syndrome	8	2.2
Cerebral palsy	7	1.9
Eating disorders	4	1.1

Note that the total number of diagnoses exceeds that of the participants because of some multiple diagnoses in the same patient.

Table 3 summarizes the PSG results comparing groups by sex, diagnosis of insomnia, ADHD and ASD. Overall, females were older than males; the periodic leg movement index and the obstructive apnoea/hypopnea index were higher in males. There were some sleep stage changes, such as a significantly decreased N3 in girls, but this is likely secondary to the natural decrease in N3 occurring during adolescence with a concomitant increase in N2. Children with insomnia had a statistically significant longer sleep latency than children without insomnia, but more N3. There was a prolonged REM latency in children with ADHD and children with ASD.

**Table 3:** Results of the univariate analysis of the polysomnographic findings in the whole group of subjects recruited in this study, sub-grouped by sex, presence of insomnia, and diagnosis of attention-deficit/hyperactivity disorder or autism. All values are expressed as mean ± standard deviation

	Sex			Insomnia			ADHD			ASD		
	Males (n = 154)	Females (n = 213)	p<	No (n = 280)	Yes (n = 50)	p<	No (n = 235)	Yes (n = 95)	p<	No (n = 291)	Yes (n = 39)	p<
Age, years	12.6 ± 3.35	14.3 ± 3.28	0.00006	13.6 ± 3.28	13.9 ± 4.07	NS	13.9 ± 3.49	13.0 ± 3.13	NS	13.7 ± 3.34	12.9 ± 3.81	NS
Total sleep time, min	421.4 ± 78.64	406.5 ± 80.25	NS	413.2 ± 79.58	408.3 ± 81.83	NS	410.0 ± 81.39	418.7 ± 75.84	NS	412.4 ± 78.61	413.1 ± 89.45	NS
Sleep latency, min	32.2 ± 42.27	32.5 ± 32.45	NS	30.6 ± 29.45	42.2 ± 63.02	0.016	33.3 ± 38.53	30.2 ± 31.66	NS	32.5 ± 35.92	31.5 ± 42.33	NS
Sleep efficiency, %	81.1 ± 15.92	80.9 ± 13.95	NS	80.9 ± 14.6	81.5 ± 15.72	NS	80.8 ± 14.45	81.4 ± 15.55	NS	81.4 ± 14.35	77.4 ± 17.28	NS
Stage N1, %	11 ± 7.08	11.4 ± 7.71	NS	11.6 ± 7.64	9.2 ± 5.98	NS	11.4 ± 7.72	10.8 ± 6.77	NS	11.2 ± 7.35	11.8 ± 8.24	NS
Stage N2, %	44.3 ± 11.05	50.3 ± 10.32	0.00013	48.1 ± 10.77	47.1 ± 12.34	NS	48.3 ± 11.19	47 ± 10.54	NS	47.9 ± 11.07	47.8 ± 10.64	NS
Stage N3, %	29.5 ± 11.25	23.7 ± 9.13	0.0034	25.4 ± 9.77	29.4 ± 13.09	0.017	25.2 ± 10.64	28 ± 9.62	NS	25.8 ± 10.35	27.8 ± 10.92	NS
Stage R latency	181.1 ± 100.6	174.8 ± 108.52	NS	179.3 ± 106.57	166.2 ± 98.11	NS	168.5 ± 103.1	199.2 ± 108	0.045	171.5 ± 99.15	221.1 ± 136.86	0.0032
Stage R, %	15.2 ± 7.25	14.5 ± 6.78	NS	14.9 ± 6.93	14.1 ± 7.25	NS	15 ± 7.23	14.2 ± 6.29	NS	15.1 ± 6.81	12.8 ± 7.87	0.022
PLMS index, n/hour	4.6 ± 8	2.6 ± 5.93	0.019	3.7 ± 7.27	1.8 ± 3.88	NS	3.3 ± 7.26	3.8 ± 5.94	NS	3.4 ± 7.04	3.2 ± 5.77	NS
Obstructive AHI, n/hour	6.6 ± 16.1	3.7 ± 6.38	0.0017	5.2 ± 12.1	3.3 ± 6.23	NS	5.3 ± 13.1	3.9 ± 5.25	NS	4.3 ± 6.88	9.3 ± 27.29	0.01
Central AHI, n/hour	1.5 ± 3.59	1.7 ± 10.14	NS	1.8 ± 8.83	0.8 ± 1.17	NS	1.5 ± 7.1	1.9 ± 10.33	NS	1.7 ± 8.67	0.9 ± 1.07	NS

Note: the total number of subjects in each comparison can be different, depending on the presence of missing information in the database.

ADHD = attention-deficit/hyperactivity disorder; ASD = autism spectrum disorder; PLMS = periodic leg movements during sleep; AHI = apnoea/hypopnea index; NS = not significant.

Table 4 shows no statistically significant differences between PSG parameters in children with anxiety, depression, and the group with combined diagnosis. Children with a diagnosis of depression were older than children with a diagnosis of anxiety. Table 4 also shows a comparison of PSG results between classes of antidepressants; the only significant difference was in REM latency, longer in children taking serotonin and norepinephrine reuptake inhibitors (SNRIs) and REM percentage (smaller in the same group). PLMS index was ≥ 5/hour in 22.1% of the total patient group, in 24.9% of children taking SSRIs or SNRIs and in 13.3% of subjects taking TCAs or atypical antidepressants (chi-square 5.29, p = 0.013).

**Table 4:** Results of the univariate analysis of the polysomnographic findings in the whole group of subjects recruited in this study, sub-grouped by psychiatric diagnosis and antidepressant treatment class. All values are expressed as mean ± standard deviation

	Psychiatric Diagnosis Group					Antidepressant Class				
	None (n = 119)	Anxiety (n = 95)	Depression (n = 33)	Depression + Anxiety (n = 83)	p<	SSRI (n = 233)	SNRI (n = 22)	TCA (n = 9)	Atypical (n = 64)	p<
Age, years	12.5 ± 3.88	12.9 ± 3.07	15.4 ± 2.34	15.4 ± 2.21	0.000001	13.7 ± 2.99	16.0 ± 2.05	14.9 ± 1.96	12.7 ± 4.59	0.000023
Total sleep time, min	411.8 ± 86.85	420.3 ± 69.81	418.4 ± 88.51	402.2 ± 76.63	NS	412.7 ± 73.11	368.2 ± 114.15	392.1 ± 65.38	426.9 ± 86.36	NS
Sleep latency, min	31.5 ± 40.21	35.7 ± 40.34	27.1 ± 22.57	32 ± 31.28	NS	32.9 ± 35.63	34.9 ± 31.1	38.5 ± 27.66	29.7 ± 43.41	NS
Sleep efficiency, %	80.1 ± 16.78	81.1 ± 12.86	78.9 ± 18.6	82.9 ± 11.71	NS	80.8 ± 14.6	80.1 ± 15.73	82.9 ± 10.82	81.2 ± 15.8	NS
Stage N1, %	10.1 ± 7.08	10.1 ± 6.04	13.6 ± 7.94	13.4 ± 8.58	NS	11.7 ± 7.59	14.4 ± 8.3	8.2 ± 3.63	9.2 ± 6.48	NS
Stage N2, %	46.6 ± 12.96	47.3 ± 9.6	50.6 ± 10.36	49.4 ± 9.41	NS	48.4 ± 9.88	53.1 ± 13.0	50.0 ± 5.39	44.5 ± 13.44	NS
Stage N3, %	28.7 ± 12.79	27.2 ± 9.06	23.3 ± 6.49	21.8 ± 7.52	NS	25.3 ± 9.41	21.1 ± 10.17	28.1 ± 4.57	29.6 ± 12.95	NS
Stage R latency	172.3 ± 109.95	185.6 ± 104.86	187.8 ± 111.92	171 ± 96.91	NS	186.3 ± 101.02	191.4 ± 128.42	120.2 ± 71.49	148.5 ± 110.68	0.045
Stage R, %	14.7 ± 7.65	15.4 ± 6.94	12.5 ± 6.34	15.2 ± 6.09	NS	14.6 ± 6.75	11.4 ± 6.72	13.7 ± 5.05	16.7 ± 7.69	0.0076
PLMS index, n/hour	3.8 ± 8.74	3 ± 5.2	4.5 ± 8.1	3 ± 4.83	NS	4.0 ± 7.73	3.7 ± 5.06	2.5 ± 6.18	1.6 ± 3.14	NS
Obstructive AHI, n/hour	5.7 ± 16.84	3.6 ± 4.34	7.5 ± 12.55	4.1 ± 5.02	NS	5.1 ± 12.91	6.3 ± 7.4	1.5 ± 1.04	4.4 ± 6.73	NS
Central AHI, n/hour	2.3 ± 9.9	1.9 ± 10.32	0.8 ± 0.9	0.6 ± 0.97	NS	1.9 ± 9.65	0.9 ± 1.66	0.5 ± 0.6	0.9 ± 1.13	NS

Note: the total number of subjects in each comparison can be different, depending on the presence of missing information in the database.

SSRI = selective serotonin reuptake inhibitor; SNRI = serotonin and norepinephrine reuptake inhibitor; TCA = tricyclic antidepressant; PLMS = periodic leg movements during sleep; AHI = apnoea/hypopnea index; NS = not significant.

## Discussion

In this study we have fulfilled our initial aims. We have determined the frequency of use of one or more antidepressants in paediatric patients referred for PSG to be one out of 10. We also identified the most common antidepressants used in children referred for PSG to be sertraline (30.5%), fluoxetine (28.1%) and trazodone (13.6%). We identified the reasons for the use of antidepressants. We found that the most common diagnosis was anxiety followed by depression, while the most common comorbid conditions were ADHD and ASD. In our study, older children had more depression while younger children had more anxiety; this matches findings in the medical literature that have demonstrated that the prevalence of depression significantly increases during adolescence [12], while the median age at onset of anxiety is 11 years [13].

And, finally, we analysed the PSG parameters found in children taking antidepressants taking into consideration other factors or covariables that can produce these effects in PSG. In total, 22.1% of patients taking an antidepressant had a PLMS index > 5/hour. It can be speculated that this could contribute to sleep disruption, and perhaps daytime consequences such as daytime sleepiness or hyperactivity[14–16]; however, this cannot be established within the framework of a cross-sectional observational study, such as this study, and therefore needs future attention. In addition, although we did not observe any significant difference in the average PLMS index among the different classes of antidepressants, as we could have expected, based on our previous studies [3, 17], both SSRIs and SNRIs were associated with a higher percentage of children with PLMS index above 5/hour (24.9%) than the other antidepressant classes (13.3%). This result might be accounted for by the fact that PLMS were scored and quantified according to the AASM criteria [11], due to the retrospective nature of this study. We cannot exclude the possibility of how the use of the more recent World Association of Sleep Medicine criteria [18], used in our previous analysis [3], could have provided different results.

In addition, the retrospective design did not allow for an analysis of the effects of antidepressants on chin tone during REM sleep because this is not a parameter that is routinely assessed in children; however, we have already reported that different types of antidepressants exert different degrees of reduction in REM sleep muscle tone atonia (resulting in increased REM sleep chin muscle tone) also in children [4, 19, 20]. REM latency has already been reported to be shorter in children with ADHD [21] and ASD [22]. We can speculate that children with ADHD and ASD may take other medications that have an additive effect on REM sleep; for example, clonidine or guanfacine can significantly suppress REM sleep [23].

It was also interesting to note that insomnia was accompanied by a prolonged sleep latency, as expected, but also accompanied by increased N3 sleep stage. This might be interpreted as an attempt to recover slow wave sleep because of the chronic sleep loss experienced by these children [24].

It is also important to underline that our statistical analysis enabled us to analyse the effects of the different factors influencing PSG parameter while taking into consideration the combined effects of the other factors and, thus, indicating the probable true independent effect of each factor. This is a clear strength of this study which was possible because of the relatively large number of subjects included.

The interrelationship between sleep and psychiatric disorders is well documented and complex, with a positive correlation between the severity of psychiatric and sleep disturbance in both children and adults [9, 10, 25, 26]. However, despite this relationship, studies using objective measures of sleep such as PSG in this population are scarce [27]. An older PSG study by Emslie et al. [28] in 20 children hospitalized with depression, compared to healthy controls, showed a pattern of sleep stages similar to that seen in adults with depression, including shortened REM latency and increased REM density and REM sleep duration [29]. In this respect, it is interesting to note that among the different classes of antidepressants assessed in our study, SNRIs and SSRIs were associated with longer REM sleep latency, and smaller percentage of this stage in the case of SNRIs. This might indicate a more effective counteraction of the PSG correlates of depression in children taking these antidepressants. However, the beneficial effects on these REM sleep findings associated with SNRIs and SSRIs in children need to be further elaborated in future studies, in order to more firmly justify the conclusions. Although improving sleep quality with cognitive behavioural interventions has demonstrated a significant positive effect on mental health [30], studies correlating changes in PSG sleep parameters and clinical changes in depression/anxiety are sparse [31].

Limitations were represented by the retrospective analysis of data, the presence of missing information, the wide age range of the subjects and the absence of a matched control group, as well as the impossibility to take into account any other type of medication that might have affected the PSG results in individual subjects.

## Conclusions

In conclusion, our findings seem to indicate that it is important to understand that intervention with pharmacological treatment of psychiatric disorders in children and adolescents can contribute to sleep alterations that may have clinical consequences. However, our data can also be interpreted as being reassuring, as many of the sleep parameters were not substantially affected by the medications. Therefore, child and adolescent psychiatrists should question about the effects on sleep (both positive and negative) after initiating therapy with antidepressant medications.

## Data Availability

The datasets generated and/or analysed during the current study are not publicly available due privacy and data protection reasons but are available from the first author on reasonable request.

## List of Abbreviations

AASM: American Academy of Sleep Medicine
ADHD: attention-deficit/hyperactivity disorder
ASD: autism spectrum disorder
N1, N2, N3: non-REM sleep stages 1, 2, and 3
PLMS: periodic limb movement during sleep
PSG: polysomnography
REM: rapid eye movement
SNRIs: serotonin and norepinephrine reuptake inhibitors
SSRIs: Selective serotonin reuptake inhibitors
